# Can assistive technology support social services during Covid-19 emergency? Barriers and opportunities

**DOI:** 10.1007/s12008-021-00836-3

**Published:** 2022-02-03

**Authors:** Laura Fiorini, Erika Rovini, Alessandra Sorrentino, Omair Khalid, Luigi Coviello, Lorenzo Radi, Lara Toccafondi, Filippo Cavallo

**Affiliations:** 1 Department of Industrial Engineering, University of Florence, Florence, Italy; 2 The BioRobotics Institute, Scuola Superiore Sant’Anna, Pontedera, Pisa Italy; 3 Department of Excellence in Robotics and AI, Scuola Superiore Sant’Anna, Pisa, Italy; 4 Umana Persone Development and Research Social Enterprise, Grosseto, Italy

**Keywords:** Covid-19, Older adults, Social professionals, Technology for active aging, Technology expectation

## Abstract

During the COVID-19 emergency, most domiciliary social services were suspended to avoid the risk of contagion, leaving older people at a greater risk of social isolation. Assistive technology has the potential to support the work of social professionals in promoting social inclusion and assistance of the older people. In this context, this paper aims to investigate the expectations of social operators toward assistive technology before and during the COVID-19 emergency. It also explores how the said emergency could guide us to implement social services in the future, including a discussion on the barriers to the adoption of assistive technologies. A total of 72 social professionals participated in this study comprising of three phases: two online questionnaires and one semi structured interview. In the first two phases, the two online questionnaires were administered before and during the COVID-19 emergency to 62 social professionals. In the third phase, 10 social workers were interviewed to discuss the results of the previous questionnaires to gain an in-depth understanding. The results highlight that the COVID-19 emergency is responsible for an increased perceived need of services involving telepresence, proposing a hybrid paradigm of assistance with both remote and in-presence assistance. Furthermore, the identified barriers to technology adoption are lack of organizational structure and ready-to-use technology. As for the facilitators for the technology adoption, social workers suggested investing in education and training of social professionals to reduce skepticism towards the usefulness of technology. The social professionals involved in this study highlight a generally positive view of technology in supporting their work. Finally, the lessons learned is also presented as a guideline for researchers in this field.

**Supplementary Information:**

The online version contains supplementary material available at 10.1007/s12008-021-00836-3.

## Introduction

While life expectancy is generally increasing worldwide, frailty is the main determinant of death, and it is strictly related to the aging process [1]. In the region of Tuscany (Italy), the average lifespan has reached 85.4 years for women and 81.3 years for men [2]. This trend raised challenges for the Italian healthcare and welfare system even before the COVID-19 outbreak, especially in relation to seniors with reduced capabilities of independent living who live without the support of their nearby family and/or a habitual relationship network. These people are at a potential risk of isolation and loss of protection by public institutions.

The World Health Organization (WHO) declared the outbreak of COVID-19 a global pandemic on March 11th, 2020. As of November 27th, 2020, more than 60 million COVID-19 cases had been reported in more than 188 countries and more than 1.42 million people had died all over the world. Italy was strongly hit by COVID-19 pandemic, reaching 245′338 total cases and 35′092 total deaths by July 2020 and 1.48 million cases with 52′028 deaths by the end of November 2020 (2nd wave) [3]. The Italian Istituto Nazionale di Statistica (ISTAT) identified people belonging to the 60 + age group as having a higher risk of death (up to 30% in male seniors) if infected by COVID-19 [4]. Additionally, specific comorbidities increase the deadly trend toward mortality [5] and a chronically ill or oncological patient has a more than doubled risk of developing symptomatic COVID-19 (up to 56%) compared to a healthy subject [6].

In March, 2020, more than 1′000 people were being tested positive each day. Consequently, the Italian government, among other countries’, imposed a lockdown to reduce the risk of contagion. Similarly, Italian citizens have been living under another “soft” lockdown starting from the beginning of November, 2020. Since November 13th, 2020, the total number of daily new cases was equal to 40′902. In these circumstances, the access to healthcare structures had become hugely limited. On one hand, patients needs outpatient rehabilitation could not be guaranteed by the service. On the other hand, an in-patient in a healthcare facility could not be visited by relatives, and, as a result, may suffer a negative psychological impact. Additionally, many domiciliary services were also suspended. Even if social distancing resulted in reduced spread of the disease, it increased social isolation, especially in older people who lived alone. Therefore, fostering remote care is of paramount importance to containing the risk of contagion while continuing the provision of health services and patient-monitoring. This need has become evident by the ongoing COVID-19 crisis, severely if not fully crippling the physical connections, and making digital inclusion a must even for a social segment, like the older adults, that has, to a large extent, been left out of this societal transformation [7].

Assistive technologies play a key role in winning this challenge from two points of view. Firstly, from older adults’ perspective, assistive technology could promote their independent living while enhancing their quality of life [8–10]. Over the last few years, literature concerning the assessment of the role of assistive technology in the life of senior citizens has underlined the existance of a generally positive attitude of senior citizens toward technology in certain domains. For instance, technology was positively associated with social engagement from women and with informal social participation for men [11]. Furthermore, home health monitoring service seems to be more acceptable when there is a clear need or use for the technology and combined with support from implementation teams with respect to health-monitoring, safety, and wellbeing [12]. Secondly, assistive technology could support the extended caregivers’ ecosystem in multiple ways to relieve their burden [13]. It allows 24/7 real-time monitoring of vital parameters, and prompt detection of emergency. It could also support the management of neurodegenerative disorders such as dementia, Parkinson’s disease, or Alzheimer’s disease [14]. Furthermore, assistive technology could provide an easier method for older people to contact their caregivers, families, and friends [15]. Additionally, assistive technology could also promote remote patient monitoring by utilizing smart homes, telecare, and artificially intelligent monitoring systems [8].

Despite these well-known advantages, assistive technology was adopted during the COVID-19 emergency only to a limited extent. Future developments of such systems require a strong cooperation between assistive technology developers and social/health professionals to exploit the multidisciplinary approach. Indeed, their active cooperation could identify needs and customize a service/product, breaking the barriers linked with the use of technology. In this context, this paper presents an iterative and in-depth analysis of the expectations elicited from a direct experience/interaction with assistive technology during the COVID-19 emergency to support the social services. The collected data were analysed to investigate how such an emergency changed and shaped future scenarios where technology and digitalization could play a central role in assisting older adults. Particularly, the presented analysis is focused on sensors and assistive robots as main technological components of services for aging well as outlined on the roadmap and in recent review papers on assistive technology [8, 10, 16].

## Study design

### Interactive methods

The international standard ISO 9241–210:2010 [16] defines Human-Centered Design (UCD) as an approach addressed to the design and the development of systems aimed to ensure interactive systems are more usable, by applying human factors and usability knowledge and methods. Indeed, one of the main goals is to create a service/product that enables the end-users to achieve their objectives as best as possible. Poor design can be translated into a frustrating experience of a certain technology [17] resulting in a strong barrier for technology adoption. It is evident that a good knowledge of user needs, as well as usability of the product, could support and improve the decision-making in product design and manufacturing. If the product will be developed on *biased* or *false* needs, it would neither be usable nor accepted. In this context, it is important to develop the solutions on a solid foundation (i.e. the needs).

One of the key stages of the UCD approach, among others, is to test the service/product with the end-user to assess the experience and analyse the interaction to eventually refine the product. The User Experience [18] is the set of perceptions and responses of a person resulting from the use of a product, system or service and it is dependent also on the context of use. It is above all a subjective evaluation that includes every user’s emotion, belief, preference, perception, physical and psychological reaction, and behaviour that occurs before, during and after the use.

Usually, the end-users are asked to be part of the process, from the design phase to the test of the final prototype. They are requested to interact with a mock-up of or with a service/product for a limited period in a controlled environment to assess the user experience. However, some questions still remain: what happens in real life when a person starts relying on such a service/product? Will the product meet the expectation of the end-users? Despite the research actions in this field, including the previously mentioned review papers that investigate and emphasize the role of assistive technology, the pandemic emergency changed the context, revealing some problems/barriers for technology adoption. Such problems can occur only after a real use in an operative context and not in a controlled environment. In this context, repetitive interactions with end-users, starting from the beginning of the product/service design, become unavoidable to facilitate their adoption in real use cases.

### Our interactive study

Recently, the COVID-19 emergency has hugely changed the landscape of traditional assistive services, raising some open questions regarding assistive technology, such as: Have the needs of older adults changed? Has the expectation of social professionals towards technology changed during the emergency? Is assistive technology ready-to-use during the pandemic emergency or there are still some barriers to its adoption? Hence, the research questions (RQs) of this study are defined as follows:

- RQ1: How did social professionals change their mind about technology before and during the COVID-19 emergency? What are the new operative scenarios where technology could make a difference?
- RQ2: What are the main limitations and barriers to technology adoption experienced by social professionals for the assistance of older adults during the emergency? Are there facilitators that could promote technology adoption?
- RQ3: How should social care providers change the way of delivering assistance to cope with the emergency?

In this context, we exploited the UCD paradigm to propose utilization of the testing phase for a real use of the technology, to collect the feedback, and to provide guidelines for shaping the technology and the service. In detail, to answer the proposed RQs, this paper presents a methodology based on three interactive phases, namely: (i) Phase 1 “definition of caregivers’ need and their expectation toward technology”; (ii) Phase 2 “feedback collection after the use of technology during the first wave of COVID-19”; and (iii) Phase 3 “refinement and discussion of the results”. Particularly, two online surveys were administered before the Covid-19 emergency and after the first wave of the pandemic, respectively, and semi-structured interviews were performed to discuss the most relevant results raised from the questionnaires, thus outlining the guidelines for future research.

The rest of this paper is structured as follows: Sect. 2 details the methodology used, including the description of the recruitment process and the experimental protocol for the data collection. It also presents the data analysis. Section 3 reports the results of the questionnaires (attitude, expectation, and future scenarios) and the outline of the interviews such as a summary of the results. Finally, Sect. 4 and Sect. 5 discuss the results, introduce the guidelines, and conclude the work.

## Methodology

### Recruitment

All the participants were recruited, on a volunteer basis, from among the social workers of Umana Persone, a Tuscany network of 10 social cooperatives involved in the Pharaon Project (GA 857188). The recruitment and the study procedures were verified by the Data Protection Officer (DPO) of Scuola Superiore Sant’Anna (Pisa). The participants received an official invitation by email from the project manager of Umana Persone to anonymously compile the questionnaires. Before starting the questionnaires, all participants were informed that their participation was entirely based on their free will and that the collected data would be aggregated and analyzed by the principal investigator of the study for scientific and research purposes. If they agreed, they could continue with the questionnaire; otherwise, they withdrew from the study. As for the interviews, the recruitment was conducted by the project manager of Umana Persone, who informed them about the purpose of the interviews and the study. Informed consent form related to undertaking the interviews (adapted to account for the COVID-19 procedures) was provided to them. If they agreed to join the study, they were contacted for the interview. At the beginning of the interview, the ethics guidance checklist (reported in Supplementary Material) was read by the interviewer to each participant. For each participant, a code generated by a randomized algorithm was associated with the related data. The association table was stored by the principal investigator of the study following the standard security procedures.

### Data collection

**Fig. 1:**
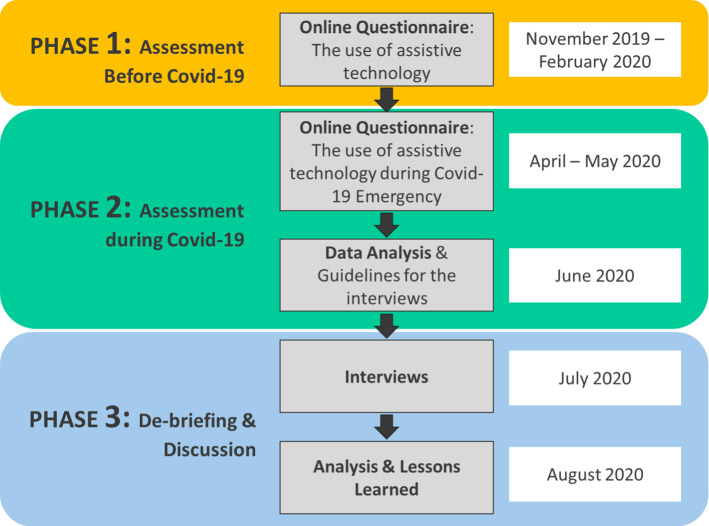
The applied methodology is based on three interactive phases: two online questionnaires to investigate the use of Assistive technology and its use during the covid-19 emergency (Phase 1 and Phase 2) and a final interview (Phase 3) that aims to discuss the questionnaires’ results and propose guidelines for future development

To accomplish the RQs of this study, the proposed methodology was mainly composed of three phases involving social professionals as depicted in Fig. 1:

- *Phase 1: assessment before Covid*-19 (B)—An anonymized questionnaire was administrated online to social operators between November 2019 and February 2020. The questionnaire was composed of four 5-item scale answers (Q1, Q2, Q3, Q4) and one open answer question (Q5). It is organized as follows: the first part is devoted to the socio-demographic information (i.e., age, range and work role) and their idea on the potential usefulness of the technology for their work (Q1), and for the elderly (Q2). The second part of the questionnaire aims to investigate their expectation toward robots (Q3) and sensors (Q4) for supporting older adults and the work of social operators. The questions were adapted from [19, 20] (as reported in Appendix I). Participants were requested to evaluate each claim on a 5-point Likert scale (from “strongly disagree” to “strongly agree”). Finally, the participants were requested to describe a scenario where technology could play a role in assisting and supporting frail people (Q5).
- *Phase 2: assessment during Covid*-19 (D)—Between April and May, 2020, social operators and professionals were requested to compile the online anonymized questionnaire for the use of technology [Q1–Q5]. A set of additional questions (Q6–Q11) were added. Q6, Q7, Q8 were multiple-choice questions, whereas Q9, Q10, Q11 were open questions. The first two items were devoted to investigating which technology the professionals would like to continue using to cope with the emergency (Q6), from among those introduced (Q7). Additionally, Q8 aims to understand what are the main limitations of technology adoption for their work (i.e., remote assistance of frail older adults). Q9, Q10 aim to investigate what are the changes requested to foster a new model to deliver services that include the technology. Finally, Q11 asks to imagine and describe a future scenario where technology could make a difference.
- *Phase 3: De-briefing and discussion*—The data collected through the questionnaires were analyzed. Based on these results, the interview guideline was prepared to discuss the results about the differences between phases B and D. Social operators were involved in this phase. Each interview lasted about 40 min and was conducted by biomedical engineers employed at Scuola Superiore Sant’Anna with experience in developing innovative services for older citizens during July 2020.

The full list of questions and the guideline for the interviews are reported in Supplementary Material. It is worth mentioning that each online survey was enriched with a glossary that briefly described each technology thus supporting the respondents (The glossary is available in the supplementary material).

### Data analysis

The survey was conducted before (B) and during (D) the COVID-19 emergency to evaluate how the emergency impacted the answers of respondents (RQ1). Namely, questionnaire D is augmented by questions that tend to explore the technological barriers experienced during the emergency (RQ2 and RQ3). Thus, the first analysis of the questionnaires relies on the comparison of the answers before and during the emergency. Mean and standard deviation values are used to assess the results of the five-Likert scale answers (Q1-Q4). The comparison is evaluated, question by question, through the statistics tool for the Mann–Whitney test provided by Python (i.e. *scipy.stats.mannwhitneyu* Python library).

Answers from multiple-choice questions (Q6, Q7) were clustered into three groups (i.e., basic, intermediate, and advanced) according to the complexity level of the selected technology. For instance, video call systems and smartphones were considered as having basic complexity; smart working and systems for health monitoring were categorized as having intermediate complexity; whereas, robots and telepresence systems were classified as having advanced complexity.

A clustering technique was used to analyze the content of open answers (Q5, Q9, Q10, Q11). Q5 was administered both in B and in D phases. Additionally, the open question Q11 was introduced in D only to investigate potential concrete applications of technologies during the emergency. Answers to these open questions were analyzed and clustered into six domains, according to the identified topics: assistance (at home or in residential facilities, physically or through a telemedicine service); telepresence; remote monitoring; models, management and digitalization (the need to deliver new health and care service models, improve digital management within the residential facility, favoring smart working); emergency/COVID-19; prevention and support (to prevent and slow down the cognitive decline). Similarly, answers to Q9 and Q10 were clustered according to the most recurrent topics, such as: internet connection issues, technology readiness, personnel training to use technology.

Finally, each interview was analysed using the method of Thematic Content Analysis (TCA) [21, 22]. The first level of coding was meant to identify themes and units of meaning. In this, we stayed close to the wording used by the respondent. In the second level of coding, we used more theoretical words. Finally, the third level of coding was the actual analysis: looking for recurring themes, coherence, and unique cases. In this paper, we reported the third level of coding, clustering the responses according to the main theme discussed.

## Results

A total of 72 participants were recruited throughout the different phases of this study. Particularly, 32 people compiled the online questionnaire on the use of technology during Phase 1 (i.e. before the COVID-19 emergency – B group). The age distribution of this group of participants varied as follows: 30–45 years old (56.26%), 45–55 years old (28.12%), and 55–65 years old (15.65%). The jobs of the participants can be summarized as follows: 8 designers, 4 heads of care services, 10 assistance coordinators, 2 members of the clinical staff (1 psychologist, 1 doctor), 1 head of human resources, and 1 social innovation manager. As for Phase 2, the questionnaire on the use of technology was compiled by 30 participants (D – group). The age distribution of the second pool of respondents varied as follows: < 30 years old (6,66%), 30–45 years old (50%), 45–55 years old (36,66%), 55–65 years old (6,66%). Their working roles are: 10 assistance coordinators, 8 designers, 7 heads of the care services, and 5 social workers. Only 23 participants filled the “During COVID-19 emergency” section (Q6-Q11). As for the interviews conducted within Phase 3, a total of 10 social workers were recruited. On average, the participants have been worked in the social assistive field for a period of 14.4 ± 7.1 years. Four of them had a managerial profile, whereas 6 subjects had a more operative profile (i.e., psychologist, social workers).

### Questionnaire: expectation toward technology

Both groups of participants (B and D) agree/strongly agree that technology can be useful for their work (Q1: m_B_ = 4.44; m_D_ = 4.47), with a slight improvement in D. Similarly, both groups of participants agree that technology can be useful for older people (Q2: m_B_ = 4.31; m_D_ = 4.06), with a small decrease of the agreement’s value in D.

Regarding the role of the robot (Q3), the Mann–Whitney test highlights several significant differences (*p* value < 0.05) among the participants’ perspectives before and during COVID-19 emergency. Among these items, the average value of the answers in B is higher than in D, reflecting a greater expectation towards the use of a robot before the emergency compared to that during the emergency. As for the role of sensors, the Mann–Whitney test highlights several significant differences (*p* value < 0.05) between participant’s expectations before and after COVID-19. The higher average value of these items in B, as compared to D, confirms the decrease in expectation towards technology after facing the emergency (the complete results are reported in Table 1).

**Table 1:** Average values and standard deviation of Q3 and Q4 items before (B) and during (D) the covid-19 emergency. For each item, the *p* value is also reported. An asterisk indicates that there are differences in that answer

Items	Q3 “I think that robot…”				Q4 “I think that sensor…”			
	Code	B Average (SD)	D Average (SD)	*p* value	Code	B Average (SD)	D Average (SD)	*p* value
Will be useful in my job	Q3.1	3.25 (1.24)	3.1 (1.21)	0.324	Q4.1	3.47 (1.48)	3.56 (1.35)	0.5
Could help and assist the older persons	Q3.2	4.18 (0.82)	3.73(0.91)	0.016*	Q4.2	4.59 (0.66)	4.36 (0.66)	0.047*
Could help and assist their family	Q3.3	4.34 (0.86)	3.8 (0.96)	0.004*	Q4.3	4.65 (0.54)	4.23 (0.77)	0.008*
Could “stole” your job	Q3.4	1.94 (1.07)	2.06 (1.17)	0.318	Q4.4	1.75 (0.98)	1.63 (0.72)	0.444
Could introduce new job opportunities	Q3.5	4.37 (0.71)	4.00 (0.64)	0.007*	Q4.5	4.06 (0.95)	3.80 (0.88)	0.092
Could reduce the need of the presence of the caregiver	Q3.6	3.12 (1.36)	2.36 (1.1)	0.012*	Q4.6	3.44 (1.19)	2.80 (1.09)	0.017*
Could increase the psychological distance between the social operator and the older person	Q3.7	2.50 (1.22)	2.50 (1.13)	0.488	Q4.7	1.78 (0.71)	1.86 (1.00)	0.488
Could help to maintain social relationship if older persons and families are distant	Q3.8	4.06 (0.88)	3.86 (0.82)	0.124	Q4.8	3.87 (1.04)	3.46 (1.04)	0.030*
Could increase the sense of security	Q3.9	4.15 (0.92)	3.73 (0.98)	0.023*	Q4.9	4.19 (0.93)	4.03 (0.61)	0.070
Could be useful in an emergency situation	Q3.10	4.47 (0.67)	4.03 (0.89)	0.015*	Q4.10	4.47 (0.72)	4.36 (0.55)	0.147
Is a negative element in the relationship between social operators and older persons	Q3.11	1.94 (1.01)	2.03 (0.80)	0.227	Q4.11	1.75 (0.88)	2.03 (1.03)	0.14

Differences in the answer are highlighted by asterisk and bold value

### Questionnaire: technology during Covid-19 emergency

**Fig. 2:**
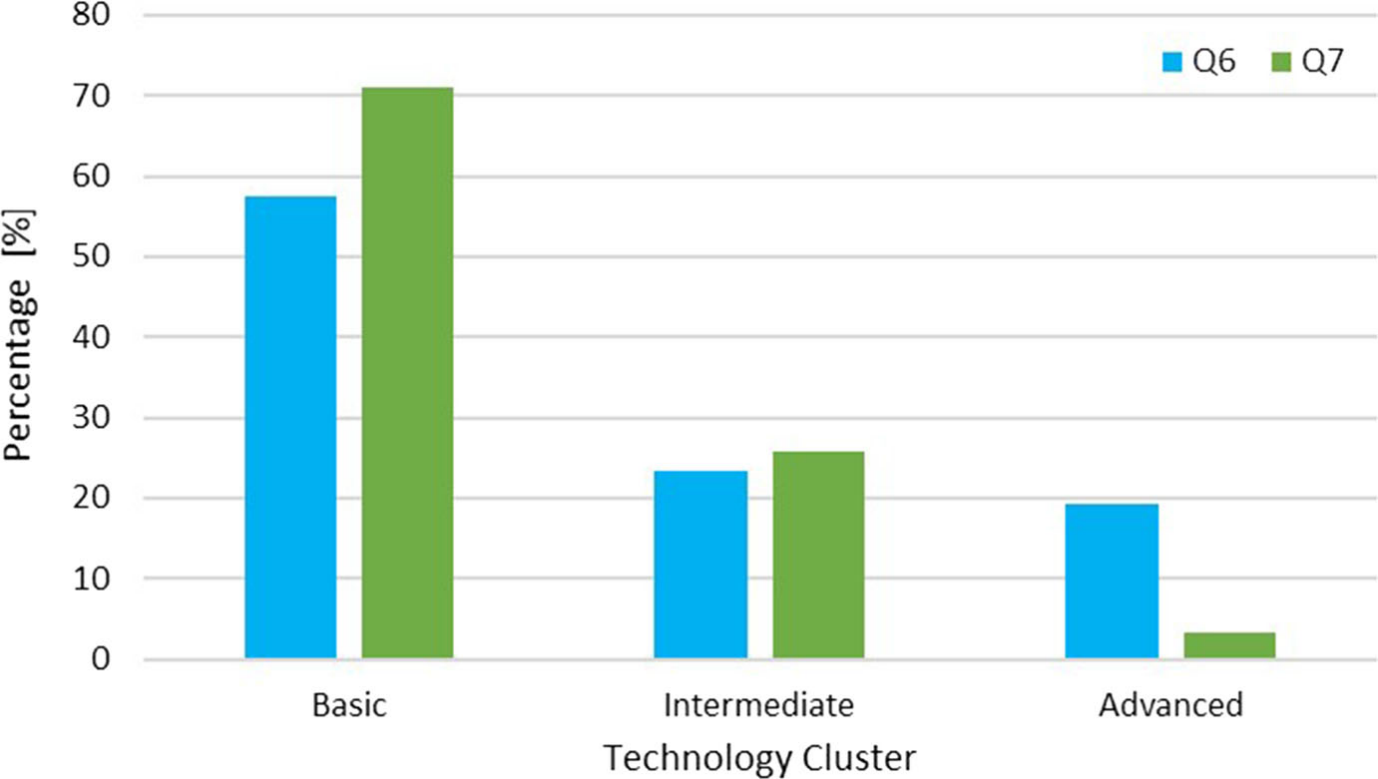
Comparison of “Technology I would like to use to cope with the emergency of Covid-19” (Q6), blue bars and “Technology I used to cope with the emergency of Covid-19” (Q7), green bars

The respondents were asked to select a maximum of 3 choices from a list of technologies in response to Q6 (“What technology I would like to use in my work to cope with the emergency I am experiencing?”). After discarding the invalid responses of 3 participants who selected more than 3 choices, a total of 47 selected choices from 20 participants were obtained. As for Q7 (“What technology have I introduced into my work to cope with the emergency?”), respondents were asked to answer without limit to the number of choices, from the same list. After discarding the invalid response of 1 participant who did not select any choice, a total of 31 selected choices were collected. The results are reported in Fig. 2. The selections related to basic technology increased from Q6 to Q7 (+ 13.52%), those related to intermediate technology remained the same, those related to the advanced group decreased (-15.92%). These results underline that there exists a desire to use advanced technology to combat with the emergency. The answers to Q7 are aligned with the national trends highlighted in [23] that show how social assistance strengthened digital skills(if already present) in the face of the emergency, modifying their way of intervention. The presented results point out that there exists an opportunity for more innovation since social operators are open to introducing advanced technology (Q6, 19.15%).

**Fig. 3:**
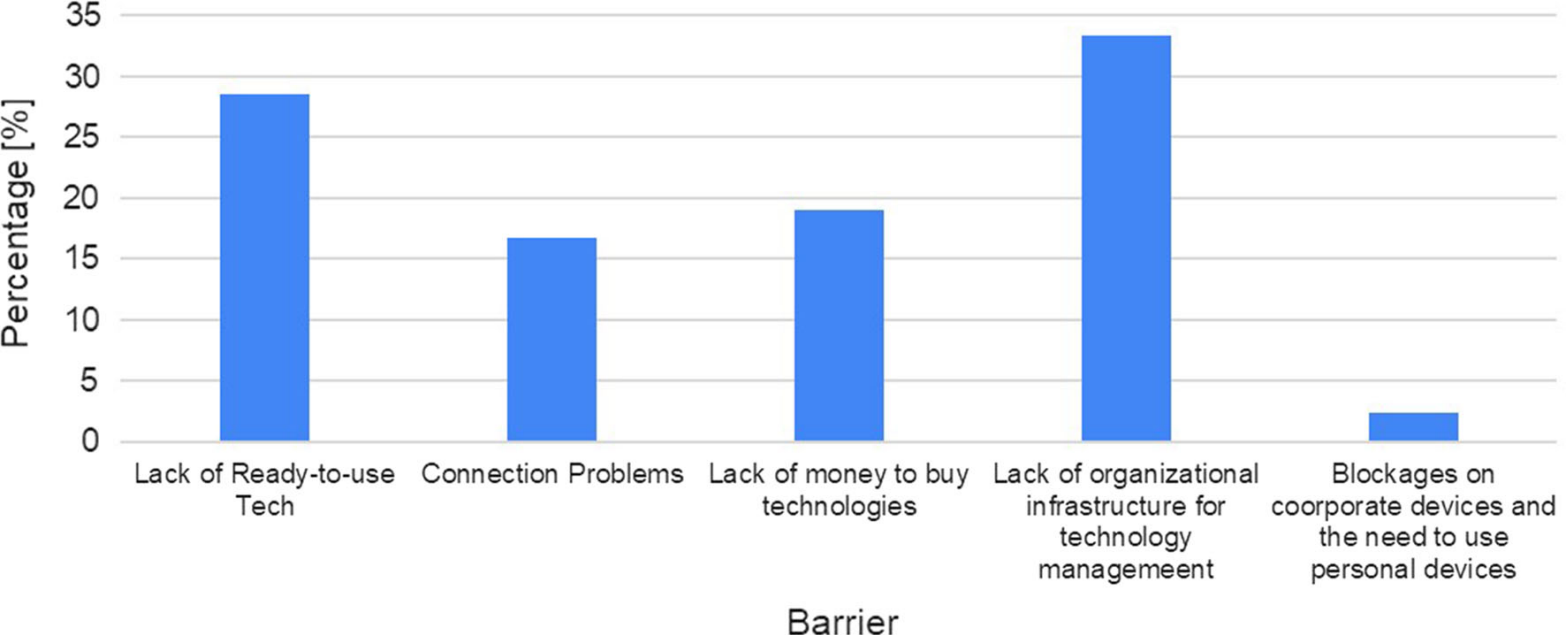
Percentage of Q8 answer (“the barriers which affected the adoption of helpful technologies during the COVID-19 emergency”)

The respondents were asked to identify, with no limit to the number of selected choices, the barriers affecting the adoption of helpful technologies during the COVID-19 emergency (Q8). Out of the 42 choices selected, the barriers were identified to be: “Lack of organizational infrastructure for technology management” (33.33%) and “Lack of ready-to-use tech” (28.57%), “Lack of money to buy technologies” (19.05%), “Internet Connection Problems” (16.67%), and “Blockages on corporate devices/Need to use personal devices” (2.38%) (see Fig. 3).

The answers to question Q9 (“What is necessary to change/update in the service’s models to include this technology?”),enable us to identify limitations that prevent the use of the proposed technology as well as some suggestions that should bepursued to foster technology adoption. Bureaucracy and internal regulations of the assistive services were repeatedly described as “marvelous” and complex. Hence, simpler procedures and regulations should be introduced and standardized to allow technology adoption. Furthermore, it was identified that the use of technology is limited by external factors, such as the need for money, and some physical restrictions related to the environment in which it will be located. Among these external factors, lack of internet connection is considered the most inhibiting. Independent of the technology, one main requirement is that it should be easy to use. Additionally, 5 respondents underlined that it is important to have reliable and ready-to-use technology for shaping future services. This will incentivize the personnel to frequently use it along with regular social services and obtain updated information online. Connected to it, one suggestion to overcome these barriers is to train the personnel on the correct usage as well as organize an awareness campaign to incentivize the use of technology (9 respondents). According to the participants, it is also important to ensure equal access to technology for people in need.

The results collected on Q10 (“Do you think that we can come back to an elderly assistance situation which is the same as the one before the emergency or it is necessary to include some changes in the way we model the services?”) highlight that the assistance service to older persons will be affected by some changes (18 participants out of 21). Discarding the comment of one participant, who did not answer the question, some of the suggested changes belong to the need of introducing technology to support assistance services as well as introducing general rules to cope with the COVID-19 emergency that can affect the delivery of the service (i.e., keeping a social distance during the services and handling visitor’s access to the structure). On the contrary, 2 participants out of 21 stated that the elderly assistance will remain the same as that before the emergency. They reported that by using the appropriate devices (i.e., Personal Protection Equipment (PPE) which includes masks and gloves), any emergency could be handled.

### Questionnaire: future scenarios

**Fig. 4:**
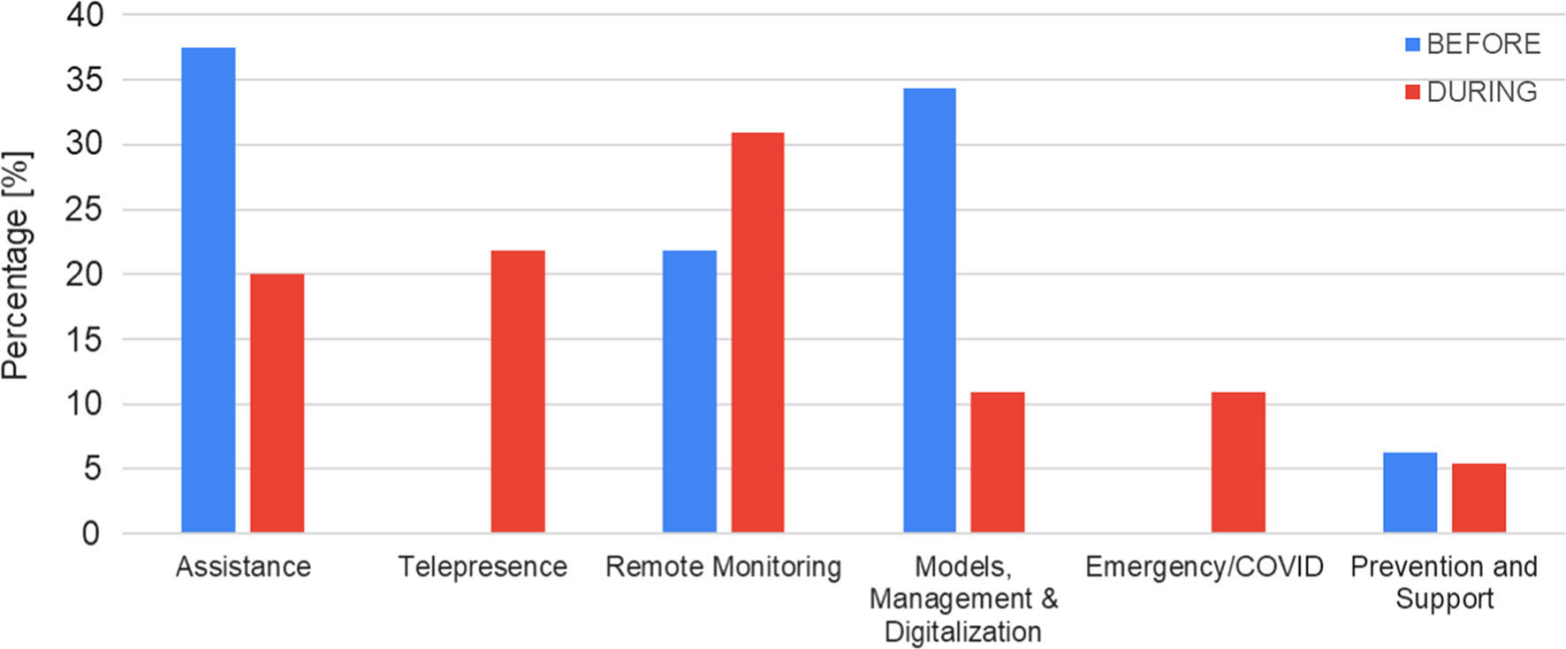
How technologies can be useful in social work: potential scenarios before (blue) and during (red) Covid-19 emergency (Color figure online)

In this section, we the answers to the questions Q5 (“Imagine a scenario where technology can be useful in your work”) (B and D) and Q11 (“Imagine a scenario where technology can be introduced within the social and health care processes”) (only D) are reported. We expected that the latter question could give us a deep insight into the former question, but, in several cases, the answer was different. Indeed, not all the people that considered the technology useful for a specific scenario, have been able to imagine the same scenario implemented within the social and health care processes. Clustered answers from these questions are reported in percentage in Fig. 4.

In Fig. 4., it is easy to identify the great impact of COVID-19 in the collective consciousness of social operators. More than 10% of respondents directly named the COVID-19 emergency in their answers as a potential scenario for technology application (Q5A and Q11). Moreover, the most relevant topic during the emergency is the identification of Telepresence as a very useful service for allowing the social workers to maintain ordinary services delivered before COVID-19. (e.g., support and companionship). Although the usefulness of Assistance decreases in D (from 37.5 to 20%), the simultaneous increase of Telepresence (21.8%) might suggest the idea of assistance simply changes, probably influenced by social restrictions, moving from the traditional, physical presence to a virtual presence. Also, the increasing interest in Remote Monitoring (+ 30%) highlights the need to monitor older people, especially alone or living in rural areas, considering the difficulties in providing physical constant support to those people. Differently, the need to prevent dementia and support for the cognitive decline has scarcely changed between B and D, reflecting a constant need of those workers that assist frail people. On the other hand, the use of technology to reshape service models or ordinary management (e.g. personnel turnover in the residential facility) has decreased in D, underlining how the emergency has changed focus to improving the services for directly supporting older people.

### Interview results and discussion

The interviews discussed some of the results achieved with the online questionnaire. Particularly, the objectives of the interviews are:

1. QI1—Understanding the barriers to technology adoption during the emergency by discussing the most three commons answers of Q8 and Q9.
2. QI2—Understanding the facilitators of technology adoption into the social assistance field (Q9).
3. QI3—Discussing the way the social operators look at the telepresence service, which was strongly suggested as a future service after the emergency (Q5 and Q11).
4. QI4—Investigating the changes in expectations of robots and sensors related to the physical presence, the older person's safety, and the support to family members (Q3).

As for the results, 9 respondents agreed with the statement that one of the limitations to the use of technology in this emergency was the lack of ready-to-use technology (QI1). It is worth mentioning that 6 respondents remarked they experienced connectivity issues during the COVID-19 emergency. From a technical point of view, social operators would like to have stable, reliable, and ease of use systems to be used in assistance services to confront the problems due to the COVID-19 related social distancing. Moreover, 8 respondents agreed that one of the limitations of the use of technology in social assistance services was the lack of an organizational infrastructure (at a cooperative level) that would facilitate the introduction of this technology. Particularly, they underlined a lack of professional figures in charge of managing and taking care of all aspects concerning the entry of technology into the working environment [“It is necessary to have someone who organizes and manages the technology and makes the operator and the user aware of its importance” (Respondent 5)].

All the respondents agreed that the two most important facilitating factors for the introduction of technology in the social assistive service models (Q12) are: (i) education toward technology and (ii) ensuring equal access to technology [“Education to technology is indispensable for both the elderly and caregiver. Operators and users have a lot of distrust and need to be educated” (Respondent 2)]. Particularly, the participants identified and discussed three main changes to introduce to the social assistive services: (i) Social cooperative managers should invest in training courses to educate and shape the mind of social professionals to foster the technology adoption and reduce the general skepticism toward technology. (ii) Reliable telepresence and remote monitoring systems should be introduced to monitor older adults 24/7. (iii) The technology introduced during the emergency to provide services should be kept since it improves the quality of assistance.

As for the telepresence service, all the respondents agreed on the important role of the technology in managing the emergency [“Technology can be very supportive and helpful in the work allowing to improve monitoring and to be timely in emergencies” (Respondent 9)]. None of the participants used to think about telepresence before the emergency, because they did not need it, and they used to organize the assistance services based on physical presence. In this sense, the COVID-19 emergency is a “disruptive-point” for healthcare services and makes evident the utility and potential of telepresence services for domiciliary assistance [“Social distance during the emergency created the need for service. Physical presence must not be replaced but improved by technology” (Respondent 1)]. Additionally, they welcome the introduction of efficient and continuous telepresence and remote monitoring services [“The introduction of telepresence services and environmental sensors and continuous monitoring of vital parameters is important” (Respondent 3)].

None of the respondents agreed with the change in the expectation concerning the security of the robot (Q3.9/Q3.10) and the use of sensors to support the family members (Q4.3) [“The change of opinion is due to a drop in expectations compared to the beginning that one had for robots and sensors” (Respondent 4). “The health emergency has generated an "abandonment anxiety" that leads to a bad opinion about everything that can keep people far away” (Respondent 5)]. The respondents confirm that, during the emergency, it was important to have functioning and stable remote assistance systems to compensate for ordinary supplied services that have been stopped during the healthcare emergency.

## Discussion and future guidelines

Social professionals see great potential in the use of technology before and during the COVID-19 as remarked by Q1 and Q2 (average answers > 4). Particularly, after the COVID-19 emergency, they slightly increased their positive view about the use of assistive technology in their work (Q1 and Q2). As reported in Table 1, there are differences in the Q3B/Q3D and Q4B/Q4D answers and the D average answers were lower than the ones collected in B. These results were deeply discussed during the interviews and the respondents did not agree with the results since they are thinking that technology could make the difference. Most notably, as remarked by the interviewed people, we found lower values because they experienced several problems with technology mainly related to the internet connection, readiness, ease of use. Before the COVID-19 they probably only know about technology, but they did not have any direct experiences. During the emergency, they had to introduce the technology in their work to cope with the emergency, and they found some limitations (barriers). Indeed, most of the respondents indicated that they introduced the technology to face the emergency and, probably, its usage modified their expectations. As remarked by Q7 compared to Q6 (Fig. 3), although social professionals would like to use more complex and advanced technologies, they only introduced and used very basic technology (i.e., smartphones, tablets, videoconference systems).

It is evident that Covid-19 emergency has led to a “fast transition” to digitalized tools like telemonitoring and telepresence services, but but these services should be re-designed to be compliant with regulations and privacy (e.g. GDPR). This emergency has modified the picture of future scenarios (Fig. 4), changing the priority to introducing new services that could promote remote assistance. It is worth mentioning that all the interviewed people remarked the importance of physical presence. Telepresence, indeed, should be an added value of assistance and not a substitution for physical presence. The remote presence should allow remote and continuous assistance even when it is not possible to meet and assist physically the older person. Indeed, all the telepresence services and the remote monitoring services proposed should lead toward a new service model where technology is used in addition to the normal services thus increasing the level and the quality of monitoring and assistance. Additionally, to foster the adoption of this assistive technology in the social care process, it should also be integrated into the socio-sanitary system, thus being used and integrated into insurance models.

Concerning the barriers and facilitators of technology adoption, the questionnaire results (Q8, Q9, Q10) and the interviews highlight that incorporation of technology into the social assistive services cannot be achieved though a “Buy&Use” paradigm and it should be reformulated into a broader process. Table 2 summarizes the important steps to consider with respect to the themes to promote the technology adoption and speed up the digitalization process. These findings could be exploited in a broader sense at a global level. A recent survey conducted between March 2020 and April 2020 by Deloitte^1^underlined that the three big challenges related to the digital transformation at Italian level are: i) “Bureaucracy” in healthcare (63.6%), “Training Staff to use technology” (46.6%) and “Cost of technology” (41.9%). Remarkably, the aforementioned results are aligned to this research. Indeed, the respondents said that one of the barriers is related to the high cost of the technology that is not always affordable. Interviews results remark that bureaucracy and a lack of organizational managerial structure that can support the digitalization problems are another limitation in the current scenario of assistance. Additionally, all the respondents remarked the necessity to educate the social operators and formal caregivers in using this technology. They need to be trained to the use of technology in their job to exploit its potentiality and benefits. Conversely, it is noted that the university coursework of healthcare givers does not currently include any course related to the use of the technology in supporting their work. In this context, changes in the educational path should be planned at a higher level, if we wish to stimulate the mind and the attitude of next generation of social workers towards the proper use of technology.

**Table 2:** Barriers, limitations, and opportunities to foster technology adoption into social assistive care

Theme	Barrier/limitations	Challenges/opportunities
Technology Readiness	Robots and sensors need to be improved in terms of ease of use Total lack of internet connection in users' homes or poor quality of connection due to geographical location Lack of technology ready to use Equity in access to technology resources	More efforts to test ready-to-use technology into real environments without the presence of technicians to highlight limitations and opportunities Promote actions to equally distribute technology on the territory included rural areas and ensure accessibility to technological services
Digitalization of processes	Distrust in technology from older adults and social professionals Too much bureaucracy to access technology	Introduce and use technology Create new Job opportunities to foster technology adoption and its correct management within the social cooperatives Enhance the quality of Job perceived Maintain the occupational level Educate social operators and users about the use of technology. Promote training sessions to facilitate the correct use of technology
New service models	Not equal access to technology Foster the introduction of technology in assistive care	Rethink the care and assistive models to include technology as key elements Rethink the co-role of public and private entities in the administration of social/assistive services Create synergies between all the stakeholder of the care process Granting equal access to technology
Ethics & Data Management	Not fully clear use of acquired data	Users should perceive that they have benefited from the acquired data because this data shall be useful to improve the services they are going to use. Indeed, older people should be data producers as well as data users Compliance with GDPR and data agreement between service and technology providers should be investigated to find the optimal way to cooperate
Open Innovation	Lack of technology managers which lead the correct management/use of technology	Co-create new digital services with different types of stakeholders to identify barriers and facilitators to promoting technology adoption and to learn from mutual experiences Change the way to think about “technology innovation”. It is not just a way to improve the service, but it could become a path to follow

## Conclusion

Over the last years, several research papers have focused on the use of technology for older adults exploring the acceptability, usability, and user experience domains among others. However, this paper is focused on analyzing and discussing what changes before and during the COVID-19 emergency in terms of assistive technology and related scenarios where technologies could play a pivot role. More than 70 socialwork professionals, from various backgrounds, were involved at different stages of the analysis. Identified current limitations and barriers in the use of technology for these professionals led to propose and promote a new vision for future services related to assistive technology. One significant contribution of this paper was to discuss the obtained results and provide for the research community some guidelines for future research and educational paths.

## Supplementary Information

Below is the link to the electronic supplementary material.

Supplementary file 1 (PDF 165 kb)

Supplementary file 2 (PDF 109 kb)

Supplementary file 3 (PDF 192 kb)

## Data Availability

Data are available on request to interested people. Send an e-mail to the correspondent author.

## Footnotes

1 “Digital Transformation: Shaping the future of European Healthcare”, available at https://www2.deloitte.com/uk/en/pages/life-sciences-and-healthcare/articles/european-digital-health.html
