# Supplementary material for: Can assistive technology support social services during Covid-19 emergency? Barriers and opportunities
Source: Int J Interact Des Manuf. 2022 Feb 3;16(1):359–70. doi: 10.1007/s12008-021-00836-3 (PMC8810343; doi:10.1007/s12008-021-00836-3)
Supplement: Supplementary file 1 — (PDF 165 kb) [file 12008_2021_836_MOESM1_ESM.pdf]

Supplementary Material

Article Title: Can Assistive Technology support social services during Covid-19 emergency? Barriers and Opportunities

Journal Name: International Journal of Interactive Design and Manufactoring

Author Names: Laura Fiorini, Erika Rovini, Alessandra Sorrentino, Omair Khalid, Luigi Coviello, Lorenzo Radi, Lara Toccafondi and Filippo Cavallo

Corresponding Author contact: Laura Fiorini, Università degli studi di Firenze, Dipartimento di Ingegneria Industriale, e-mail: laura.fiorini@unifi.it

Interview Guidelines

Code Respondent:	☐ 01 ☐02 ☐ 03 ☐ 04 ☐ 05 ☐ 06 ☐ 07 ☐ 08 ☐ 09 ☐ 10
Work	Which is your work role?
Year of work	How long have you been dealing with you work? (in years) ________

Dear Mr./Mrs:………………………………….,

Thank you for accepting to participate in this interview for discussing the results of the questionnaire. Your collaboration is appreciated! I will shortly explain the interview procedures:

- This interview aims to discuss the adoption of technology during covid-19 emergency starting from the results of an online survey.
- This data may be used for future publications including, but not limited to: academic journals, websites and policy papers.
- The interviewer/researcher conducting this interview will make sure that the data is treated confidentially, and that the data will not be traceable to a specific individual to safeguard privacy.
- If you do not feel comfortable to give answers to a question, you can state this without providing any reason and the interviewer/researcher will skip the question.
- If you do not feel comfortable to continue the interview any further, you may at any moment discontinue the interview without providing any reasons.

Do you have any questions? Do you understand and accept the above mentioned procedures? If so, we will start the interview.

QI1: Understanding the barriers on technology adoption by discussing the most three commons answers of Q8 and Q9.

1. 12 people responded that one of the limitations to the use of technology in this emergency was the lack of ready-to-use technology, do you agree?
    a. If YES
        i. what expectations did you have for the technology?

b. If NO,
   i. what is your experience?
   ii. How long have you been able to use technology in your work?
2. 14 respondents said that one of the limitations was the lack of an organizational infrastructure (at cooperative level) that would allow the introduction of this technology. Do you agree with this statement? Do you consider it a barrier to the introduction of such technology?
   a. If YES,
      i. could you better explain the shortcomings you have highlighted? How could the situation be improved to enable the technology to be adopted?
   b. If NO,
      i. how did you manage to introduce the technology? What would you recommend to other people who have had this problem to allow the technology to be used?
3. Have you had connectivity issues?

QI2 - Understanding the facilitators of the technology adoption into the social assistance field (Q9).

4. The results of the questionnaire underlines as three points to work on to facilitate the entry of technology into your field of operation: the technology improvement (5 votes); the technology education (9 votes); the ensuring access to technology (5 votes). Do you agree?
   a. If YES,
      i. What was your experience?
      ii. Would you give me a few more details?
   b. If NO,
      i. what change should be made to encourage the adoption of technological solutions in this crisis context?
   c. What is the biggest change that care services will have to introduce for post-Covid?

QI3 – Discussing the way the social operators see the telepresence service, that was significantly introduced as future service after the emergency (Q5 and Q11).

5. The covid-19 emergency has predominantly introduced the telepresence service for future application as part of the care of the frail and lonely elderly.
   a. How do you see the telepresence service before and after covid-19 emergency?
   b. According to the results, there was an increase need in this service. Do you agree?
   c. Why was this need not so predominant before?
   d. Were you already using telepresence services before covid-19 emergency?

QI4 – Investigating the changes into expectations of robots and sensors related to the physical presence, the older person safety and the support to family members (Q3).

6. The perception of the need for the physical presence of the caregiver has changed for both robots and sensors. Before covid-19 people were much more in agreement with this statement rather than after covid-19.
   a. What is your opinion about the need for physical presence?
   b. How can technology support you?
7. As for the statement "I think the robot can improve the safety of the elderly", the average answer has gone from "very agree" to "agree"; what do you think about this change of opinion?

8. As for the statement: "I think sensors can help family members", the average answer went down from "very agree" to "agree". How could you interpret this change of opinion?
