# Supplementary material for: Can assistive technology support social services during Covid-19 emergency? Barriers and opportunities
Source: Int J Interact Des Manuf. 2022 Feb 3;16(1):359–70. doi: 10.1007/s12008-021-00836-3 (PMC8810343; doi:10.1007/s12008-021-00836-3)
Supplement: Supplementary file 2 — (PDF 109 kb) [file 12008_2021_836_MOESM2_ESM.pdf]

Supplementary Material

Article Title: Can Assistive Technology support social services during Covid-19 emergency? Barriers and Opportunities

Journal Name: International Journal of Interactive Design and Manufactoring

Author Names: Laura Fiorini, Erika Rovini, Alessandra Sorrentino, Omair Khalid, Luigi Coviello, Lorenzo Radi, Lara Toccafondi and Filippo Cavallo

Corresponding Author contact: Laura Fiorini, Università degli studi di Firenze, Dipartimento di Ingegneria Industriale, e-mail: laura.fiorini@unifi.it

Glossary

[ ] Robot

I robot sono oggetti elettronici che aiutano a svolgere compiti che per gli esseri umani sono difficili, pericolosi, oppure molto ripetitivi. Per esempio, i robot nelle fabbriche aiutano gli uomini a costruire le automobili. Alcuni robot, che somigliano nel loro aspetto agli esseri umani, possono essere usati per aiutare i medici e gli infermieri ad assistere le persone che ne hanno bisogno o per aiutare le persone stesse a compiere alcune attività quotidiane come quelle di pulizia degli ambienti.

[ ] Internet of Things (Rete delle Cose)

Tutto ciò che si può collegare ad internet fa parte dell'Internet of Things: dal semplice computer ad un cellulare, un termometro, una lampadina intelligente o anche il termostato dell'impianto di riscaldamento. Grazie ad internet questi dispositivi possono "dialogare" tra di loro. È possibile, per esempio, tramite il cellulare, accendere o spegnere una lampadina o misurare la temperatura presente in casa mentre si fuori.

[ ] Intelligenza artificiale

È la capacità dei computer o dei robot di fare ragionamenti o prendere delle decisioni. Computer e robot cercano di imitare quello che fa l'intelligenza degli uomini e sono addirittura in grado di imparare a risolvere problemi semplici o, in alcuni casi, anche molto difficili. Un robot con intelligenza artificiale è per esempio il robot aspirapolvere che si trova in molte abitazioni moderne. Infatti, anche se il robot non conosce com'è fatta la casa, riesce a pulirla con ottimi risultati.

[ ] App, dispositivi mobili e servizio di monitoraggio.

Le App sono le applicazioni e funzionalità presenti nei cellulari moderni, chiamati smartphone. Vengono chiamati generalmente dispositivi mobili in quanto possono essere facilmente trasportabili, ad esempio in tasca. Grazie alle app i cellulari riescono a fare molte altre cose in aggiunta alle telefonate. Ad esempio, ordinare una pizza, mandare dei messaggi agli amici, visualizzare una mappa, scattare delle fotografie. Sulle app puoi ricevere anche informazioni sullo stato di salute dei tuoi genitori anziani o dei tuoi nonni riuscendo così a monitorare il loro stato di salute anche a distanza.

[ ] Sensori e Dispositivi indossabili

Esistono alcuni dispositivi elettronici che possono essere indossati dalle persone e che misurano alcune caratteristiche del corpo umano. Per esempio, ci sono braccialetti che misurano i battiti del cuore o il numero di passi fatti in una giornata, oppure anche la qualità del sonno o la pressione cardiaca. Di solito si collegano con i cellulari a cui trasmettono le informazioni che registrano.

[ ] Telepresenza

Con il covid-19 abbiamo sperimentato la distanza fisica, il non poter andare a trovare di persona i nostri cari o le persone di cui ci occupiamo. Alcune tecnologie, chiamati robot per la telepresenza, possono supportarci nella telepresenza, ovvero ad essere presente e muoverci in un ambiente anche quando siamo fisicamente distanti.

ENGLISH

[ ] Robots

Robots are manufacturers that help perform tasks that are difficult, dangerous, or very repetitive for humans. For example, robots in factories help humans build cars. Some robots, which resemble humans in their appearance, can be used to help doctors and nurses assist people who need them or to help people themselves perform some daily tasks such as cleaning rooms.

[ ] Internet of Things

Anything that can be connected to the internet is part of the Internet of Things: from a simple computer to a cell phone, a thermometer, a smart light bulb or even the thermostat on your heating system. Thanks to the internet, these devices can "talk" to each other. You can, for example, through the cell phone, turn on or off a light bulb or measure the temperature in the house while you are out.

[] Artificial intelligence

It is the ability of computers or robots to make reasoning or decisions. Computers and robots try to imitate what human intelligence does and are even able to learn how to solve simple or, in some cases, very difficult problems. A robot with artificial intelligence is, for example, the robot vacuum cleaner found in many modern homes. In fact, even if the robot does not know what the house looks like, it can clean it with excellent results.

[ ] Apps, mobile devices and monitoring service.

Apps stands for applications found in modern cell phones, called smartphones. They are generally called mobile devices because they can be easily carried around, for example in a pocket. Thanks to apps, cell phones are able to do many other things in addition to phone calls. For example, order a pizza, send messages to friends, view a map, take pictures. On apps you can also receive information about the health of your elderly parents or grandparents and monitor their health from a distance.

[] Wearable sensors and devices

There are some electronic devices that can be worn by people that measure certain characteristics of the human body. For example, there are bracelets that measure heartbeats or the number of steps taken in a day, or even sleep quality or heart pressure. They usually connect with cell phones to which they transmit the information they record.

[] Telepresence

With covid-19 we have experienced physical distance, not being able to physically visit our loved ones or the people we care for. Some technologies, called telepresence robots, can support us in telepresence, that is, to be present and move in an environment even when we are physically distant.
