# Supplementary material for: Can assistive technology support social services during Covid-19 emergency? Barriers and opportunities
Source: Int J Interact Des Manuf. 2022 Feb 3;16(1):359–70. doi: 10.1007/s12008-021-00836-3 (PMC8810343; doi:10.1007/s12008-021-00836-3)
Supplement: Supplementary file 3 — (PDF 192 kb) [file 12008_2021_836_MOESM3_ESM.pdf]

Supplementary Material

Article Title: Can Assistive Technology support social services during Covid-19 emergency? Barriers and Opportunities

Journal Name: International Journal of Interactive Design and Manufactoring

Author Names: Laura Fiorini, Erika Rovini, Alessandra Sorrentino, Omair Khalid, Luigi Coviello, Lorenzo Radi, Lara Toccafondi and Filippo Cavallo

Corresponding Author contact: Laura Fiorini, Università degli studi di Firenze, Dipartimento di Ingegneria Industriale, e-mail: laura.fiorini@unifi.it

Questionnaire on the use of technology

Before and After Covid-19

INFORMED CONSENT

We propose you to fill in the following questionnaire on this Google Form platform in order to investigate the expectation toward social robots and sensors such as to investigate suitable future scenarios. Participation in the questionnaire is anonymous and voluntary.

The answers, elaborated in aggregate form by the BioRobotics Institute of Scuola Superiore Sant'Anna, by the team coordinated by Prof. Filippo Cavallo (filippo.cavallo@santannapisa.it), can in no way be linked back to your identity.

This data may be used for future publications including, but not limited to: academic journals, websites and policy papers

If you agree with this statement, please click on “continue”.

First part: Socio demographic information

SD1 - Age Range

1) <30 2) 31-45 3)46-55 4) 56-65 5) <66

SD2 - What is my job?

Second part: Expectation toward technology

Q1: I think that technology can be useful for my work

1) Strongly Disagree 2) Disagree 3) Neither Agree nor Disagree 4) Agree 5) Strongly agree

Q2: I think that technology can be useful for elderly

1) Strongly Disagree 2) Disagree 3) Neither Agree nor Disagree 4) Agree 5) Strongly agree

Q3: I think that robot…[evaluate the sentences]

Code	Items	Strongly Disangree	Disagree	Neither agree nor disagree	Agree	Strongly agree
Q3.1	Will be useful in my job					
Q3.2	Could help and assist older person					
Q3.3	Could help and assist their family					
Q3.4	Could "stole" your job					
Q3.5	Could introduce new job opportunities					
Q3.6	Could reduce the need of the presence of the caregiver					
Q3.7	Could increase the psychological distance between the social operator and the older person					
Q3.8	Could help to maintain social relationship if older persons and families are distant					
Q3.9	Could increase the sense of security					
Q3.10	Could be useful into emergency situation					
Q3.11	Is a negative element in the relationship between the social operators and the older persons.					

Q4: I think that sensors…[evaluate the sentences]

Code	Items	Strongly Disangree	Disagree	Neither agree nor disagree	Agree	Strongly agree
Q4.1	Will be useful in my job					
Q4.2	Could help and assist older person					
Q4.3	Could help and assist their family					
Q4.4	Could "stole" your job					
Q4.5	Could introduce new job opportunities					
Q4.6	Could reduce the need of the presence of the caregiver					
Q4.7	Could increase the psychological distance between the social operator and the older person					
Q4.8	Could help to maintain social relationship if older persons and families are distant					

Q4.9	Could increase the sense of security					
Q4.10	Could be useful into emergency situation					
Q4.11	Is a negative element in the relationship between the social operators and the older persons.					

Q5: Think about a possible scenario in which the technology could support your work

[Section to fill only after the covid-19 emergency]

Third part: Technology during covid-19 emergency

Q6 - What technology I would like to use in my work to cope with the emergency I am experiencing (choose a maximum of 3 options)?

[Telepresence System & Video Call; Smartphone/Tablet; Physiological parameter monitoring system; Robot; Robotic Sanification; Remote Monitoring of person positive to Covid-19; sensors, other]

Q7 - What technology have I introduced into my work to cope with the emergency?

[Telepresence System & Video Call; Smartphone/Tablet; Physiological parameter monitoring system; Robot; Robotic Sanification; Remote Monitoring of person positive to Covid-19; sensors, other]

Q8 - What are the barriers I experienced during the Covid-19 emergency for the introduction of technologies? [Choose one or more option]

[Lack of ready-To-Use Technology; Difficulty in internet connection; Lack of money to buy technology; Lack of organizational infrastructure for the correct management of technology; other]

Q9 – What is necessary to change/update in the service's models so that to include this technology?

Q10 - Do you think that we can come back to an elderly assistance situation which is the same of the one before the emergency or it is necessary to include some changes in the way we model the services?

Q11 - Imagine a scenario where technology can be introduced within the social and health care processes
